# The cortisol response in parents staying with a sick child at hospital

**DOI:** 10.1002/nop2.245

**Published:** 2019-02-13

**Authors:** Charlotte Angelhoff, Ulla Edéll‐Gustafsson, Evalotte Mörelius

**Affiliations:** ^1^ Department of Social and Welfare Studies, Division of Nursing Sciences Linköping University Norrköping Sweden; ^2^ Department of Pediatrics and Department of Clinical and Experimental Medicine Linköping University Linköping Sweden; ^3^ Department of Medicine and Health Sciences, Division of Nursing Science Linköping University Linköping Sweden

**Keywords:** child, family nursing, hospitalized, paediatric nursing, parent, stress

## Abstract

**Aim:**

To study the cortisol response in parents staying with their child in paediatric wards, to compare the parents’ cortisol levels between the paediatric ward and at home 4 weeks after discharge and to compare the parents’ cortisol levels with data of an adult reference population, reported by Wust et al., as there are few studies investigating parental cortisol.

**Design:**

This study has a descriptive and prospective comparative design.

**Method:**

Thirty‐one parents participated. Saliva samples were collected in the paediatric ward and 4 weeks later at home.

**Results:**

The parents had lower morning awakening cortisol levels in the paediatric ward than at home after discharge. There were no statistically significant differences in postawakening cortisol or cortisol awakening response (CAR). The child's age, diagnosis or previously diagnosed chronic condition did not affect the parents’ cortisol levels. The morning and postawakening cortisol levels were lower than those of the reference population.

**Conclusion:**

The hospital stay with a sick child affects parents’ cortisol levels. Parental stress needs more attention to find interventions to prevent the risk of stress‐related complications that subsequently can affect the care of the child.

## 1 INTRODUCTION

Many children are admitted to hospital due to a temporary reduction of their health status, for example acute infections, orthopaedic conditions and acute surgery. To protect the sick child from separation, many hospitals offer accommodation for parents at the ward to enable them to be with their sick child around the clock. Being together with one's family is not only beneficial for the child's welfare as it reduces the stressful aspects of being admitted to hospital (Coyne, Hallström, & Söderbäck, 2016; Feeg et al., 2016), but has also been reported to improve parents’ sleep and mood (Angelhoff, Edéll‐Gustafsson, & Mörelius, 2018). However, worries about the sick child, uncertainty and a feeling of powerlessness are stressors that affect the parents’ ability to understand information, make the right decisions regarding the child's care and provide care for the child (Edéll‐Gustafsson, Angelhoff, Johnsson, Karlsson, & Mörelius, 2015; Stremler, Dhukai, Wong, & Parshuram, 2011).

Stressors increase the level of cortisol. Emotional draining stressors activate the hypothalamic–pituitary–adrenal (HPA) axis, which regulates cortisol release (McEwen, 2008). Saliva cortisol is an established biomarker of the stress response and reflects the biologically active fraction of cortisol. Normally, awakening in the morning causes a phasic activation of the HPA axis with a pronounced increase (38%–75%) in the cortisol levels between the morning awakening cortisol level and the postawakening cortisol level 25 min later. This effect is known as the cortisol awakening response (CAR). The HPA axis stress response is designed to help the individual to cope with stressors (Herman et al., 2016). However, a chronic activation of the HPA axis damages the cardiovascular system. Over time, this may result in stress‐related disorders such as stroke and heart attack and reduce the capability of handling stress‐related demands (Lupien, McEwen, Gunnar, & Heim, 2009; Slavich, 2016). Moreover, the HPA reactivity is also associated with the limbic system, which is central for mood and stress regulation (Elder, Wetherell, Barclay, & Ellis, 2014; McEwen, Eiland, Hunter, & Miller, 2012). An increase in the cortisol levels has a damaging effect on the hippocampus (Elder et al., 2014), which affects memory and cognitive function, such as new learning, attention and executive functions (Lupien et al., 2009; Oken, Chamine, & Wakeland, 2015).

To evaluate the activity of the HPA axis, cortisol has been studied in parents previously in different contexts. A correlation was found in cortisol between preterm infants' and their mothers when receiving family‐centred care (Mörelius, Broström, Westrup, Sarman, & Örtenstrand, 2012) and when the parents practiced skin‐to‐skin contact (Mörelius, Örtenstrand, Theodorsson, & Frostell, 2015). Low CAR in mothers of children with cerebral palsy correlated with poor health‐related quality of life (Bella, Garcia, & Spadari‐Bratfisch, 2011) and parents of children with autism spectrum disorders have low morning cortisol levels associated with high levels of stress for a long period of time (Ruiz‐Robledillo, De Andres‐Garcia, Perez‐Blasco, Gonzalez‐Bono, & Moya‐Albiol, 2014; Wong, Mailick, Greenberg, Hong, & Coe, 2014). However, no previous study has examined salivary cortisol in parents who remain around the clock at hospital with their sick child.

Higher stress and negative emotions during a day are associated with lower morning cortisol awakening levels and lower CAR on the following day. There is, thus, a smaller difference between the morning cortisol level and the postawakening cortisol level (Proulx, Klee, & Oken, 2017; Wong et al., 2014). Having a sick child in need of medical care affects the parents’ mood (Angelhoff, Edéll‐Gustafsson, & Mörelius, 2015) and mothers have reported feeling less in control during accommodation at the paediatric ward (Angelhoff et al., 2018). Therefore, our hypothesis was that parents of sick children are exposed to stressors that lead to lower morning cortisol levels and lower CARs in the hospital than those that occur at home after discharge and that they are lower than the levels in a reference population.

## 2 AIMS

The aim was to study the cortisol response (morning awakening, postawakening and CAR) in parents staying with their sick child in paediatric wards and to compare the parents’ cortisol levels at the paediatric ward and at home 4 weeks after discharge. A further aim was to compare the parents’ cortisol levels with those of a reference population.

## 3 DESIGN

This study had a descriptive and prospective comparative design. It is part of a larger project studying sleep, mood and stress in parents staying overnight at hospital with their sick child (Angelhoff et al., 2018).

## 4 METHODS

### 4.1 Participants and procedure

A sample of parents accommodated with their child in six paediatric wards at four hospitals in south‐eastern Sweden participated. Inclusion criteria were parents able to read and speak Swedish, staying overnight at their child's bedside at a paediatric ward, regardless of the child's age and diagnosis. Data were collected between September 2013–October 2015 from the same parents twice: after being accommodated at least one night in the paediatric ward and at home 4 weeks after discharge. Based on the morning awakening cortisol levels, a sample size consisting of 31 parents reaches a power >80%, effect size Cohen's d 0.25, *p *< 0.05.

As CAR on workdays differs from that on weekend days (Kunz‐Ebrecht, Kirschbaum, Marmot, & Steptoe, 2004), the nursing staff recruited all parents on Tuesday afternoons with intermissions in June–August and December, to avoid possible CAR variation during weekends, public holidays and vacations. After giving their informed consent, the parents were asked to complete a questionnaire with demographic data and were given oral and written instructions on how to take their own saliva samples directly on waking on Wednesday morning (the morning awakening cortisol) and 25 min later (the postawakening cortisol). The procedure was repeated 4 weeks later at home. To collect the saliva samples, Salimetrics oral polymer swabs and tubes were used. The parents were instructed to report the actual date and time of sampling and not to eat, drink, smoke or brush their teeth until after the sampling, as it may lowering the pH in the saliva and increase bacteria growth (Hansen, Garde, & Persson, 2008; Schwartz, Granger, Susman, Gunnar, & Laird, 1998).

The saliva samples collected at home were returned to the first author in prepaid envelopes. On arrival, the saliva samples were centrifuged and stored at −80ºC until analysis. A commercial enzyme immunoassay method was used to analyse the saliva cortisol (Salivary Cortisol Enzyme Immunoassay Kit, Salimetrics LLC, PA, USA). The inter‐assay coefficient of variation was 10% for 2 nmol/L and 6% for 30 nmol/L.

The morning awakening cortisol, postawakening cortisol, CAR and a cortisol index were considered measures of the cortisol response. The cortisol index, calculated as the CAR divided by the morning awakening cortisol, was used to adjust for inter‐individual differences. By creating the cortisol index, it was not necessary to exclude potential outliers related to individual biological differences. A change greater than 10% between the morning awakening and postawakening cortisol levels was considered as a difference in the CAR. Reference population mean values were based on data from four combined studies, including a total of 509 healthy adults with a mean age of 37.3 years (*SD* 13.6; Wust et al., 2000).

### 4.2 Statistical analysis

The statistical software program SPSS version 24 was used for data analysis. *p*‐Values < 0.05 were considered to be statistically significant. The independent *t *test compares means between two unrelated groups on the same continuous, dependent variable and was used to compare morning awakening cortisol and postawakening cortisol between the parents and the reference population. As data from the study were non‐parametric, Wilcoxon signed‐rank test was used for pairwise comparisons between the parents in the paediatric ward and after discharge at home. A Mann–Whitney *U* test was used to study possible differences in the morning awakening cortisol, the postawakening cortisol and the CAR between mothers and fathers, between parents of a child with a previously diagnosed chronic condition and parents of a child without such a diagnosis and between parents who stayed for more than one night and parents for whom the sampling occasion was their first night. Moreover, a stepwise regression method using ANOVA was used. The morning awakening cortisol, the postawakening cortisol and the CAR were set as the dependent variable in three separate models to study the effect of the independent variables including age, child's age and the child's diagnosis, on the parents’ morning awakening cortisol, postawakening cortisol and CAR in the paediatric ward.

### 4.3 Ethics

The study was approved by the Regional Committee for Medical Research (DNR 2011/1631) and performed in accordance with the Declaration of Helsinki (World Medical Association, 2013).

## 5 RESULTS

### 5.1 Participants

Thirty‐six parents participated in the study; five parents were excluded since two or more saliva samples were missing. The results consist of measurements from 31 parents (average age 39 years, *SD* 8.0). Demographic data are presented in Table 1. The children (average age 7.6 years, *SD* 5.3, range: 0–16 years) were divided into four diagnosis groups: oncology (*N = *4); surgery/orthopaedic (*N = *12); respiratory infections (*N = *8); and other conditions (*N = *7). Eleven of the participating parents had a child with a previously diagnosed chronic condition, which could be minor or major health problems: four of these children had oncological diagnoses, five children had been admitted due to surgical health conditions and two children had been admitted for other conditions (such as unspecified infection or abdominal pain).

**Table 1:** Demographic characteristics (*N* = 31)

	*N*	(%)
Marital status		
Married/cohabitant	27	(87.1)
Single	3	(9.7)
Living apart	1	(3.2)
Highest education level		
Compulsory/upper secondary school	11	(35.5)
University	20	(64.5)
Been to the hospital with the child before		
Never/once	11	(35.5)
2–10 times	13	(41.9)
>10 times	7	(22.6)
The child’s diagnosis		
Oncology	4	(12.9)
Treatment	3	(9.7)
Complication after treatment	1	(3.2)
Surgery/orthopaedic	12	(38.7)
Foot/knee	4	(12.9)
Back/spine	5	(16.1)
Jaw/throat	3	(9.7)
Respiratory infections	8	(25.8)
Respiratory syncytial virus (RSV)	3	(9.7)
Pneumonia	3	(9.7)
Trouble breathing	2	(6.4)
Other conditions	7	(22.6)
Unspecified infections/fever	3	(9.7)
Diabetes type I—new onset	1	(3.2)
Seizures	1	(3.2)
Concussion	1	(3.2)
Abdominal pain	1	(3.2)

### 5.2 Parents’ cortisol response

**Figure 1:**
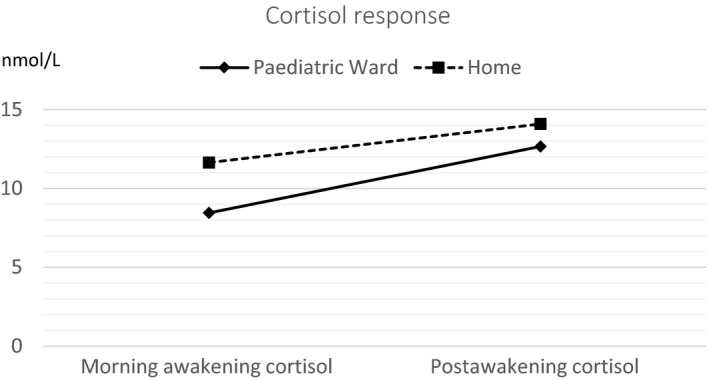
Parents’ salivary cortisol response in the paediatric ward and at home

Of the 124 collected saliva samples, 122 samples (98%) were included in the analysis. Two postawakening cortisol samples taken at the hospital were excluded due to an insufficient amount of saliva; however, the morning awakening cortisol sample from these parents was included in the analysis. Salivary cortisol levels are presented in Table 2. The parents had significantly lower (*p =* 0.01) morning awakening cortisol levels in the paediatric ward than at home (Figure 1, Table 2). In the paediatric ward, the cortisol levels had a mean increase of 71% between the morning awakening cortisol and the postawakening cortisol. At home, the cortisol levels had a mean increase of 56%. The difference was not statistically significant. In the paediatric ward, 23 parents had an increase in CAR greater than 10%, while CAR decreased greater than 10% from baseline for five parents. At home, 18 parents had an increase in CAR greater than 10% and CAR decreased by greater than 10% from baseline for eight parents. Of the parents with decreasing CAR, four parents had a decreasing CAR both at the paediatric ward and at home. There were no statistically significant differences in morning awakening, postawakening or CAR between mothers and fathers, between parents of a child with a previously diagnosed chronic condition and parents with previously healthy children, or between parents who had been accommodated for more than one night and parents staying their first night. The parents’ age, child's age or the child's diagnosis did not have any effect on the cortisol levels (morning awakening, postawakening or CAR). The reference value mean for morning awakening cortisol is 15.1 nmol/L (*SD* 6.3) for morning awakening cortisol and 23.0 (*SD* 9.1) for postawakening cortisol in adults (Wust et al., 2000). The parents’ morning and postawakening cortisol levels both in the paediatric ward and at home were significantly lower (*p* < 0.05) than those of the reference population (Table 2).

**Table 2:** Pairwise comparison of the parents’ cortisol responses

	*N*	In the paediatric ward				At home after discharge				*p*‐Value^a^
		Mean	(*SD*)	Median	(*Q*_1_, *Q*_2_)	Mean	(*SD*)	Median	(*Q*_1_, *Q*_2_)	
Salivary cortisol levels^b^										
Morning awakening cortisol	31	8.45	(3.71)	7.35	(5.48, 11.93)	11.64	(6.97)	10.05	(6.23,15.00)	<0.01
Postawakening cortisol	29	12.66	(6.13)	11.70	(7.80, 15.85)	14.09	(6.51)	14.00	(8.70, 18.25)	0.17
Cortisol awakening response (CAR)	29	4.34	(5.5)	4.00	(1.20, 7.95)	2.80	(5.42)	2.00	(−0.95, 5.25)	0.14
Cortisol index	29	0.71	(0.81)	0.51	(−0.19, 0.95)	0.60	(1.10)	0.20	(−0.12, 0.60)	0.12

a Wilcoxon signed‐rank test.

b nmol/L.

## 6 DISCUSSION

Our hypothesis was partly confirmed: the parents had lower awakening cortisol levels at the hospital than they had at home and lower levels than those of the reference population. The lower awakening cortisol in the paediatric ward may be explained by the parents having experienced acute stress the previous day. Appraisals of high stress are associated with an increase in cortisol levels during the day of exposure to a stressful event, followed by lower secretion the following morning (Gartland, O'Connor, Lawton, & Bristow, 2014; Proulx et al., 2017). Another stressor could have been waiting, which has been described the most stressful aspect of the hospital experience for family members, with a constant struggle to maintain a balance between negative and positive thoughts (Trimm & Sanford, 2010), anxiety, stress and fear (Corsano, Majorano, Vignola, Guidotti, & Izzi, 2015). Surprisingly, the awakening cortisol levels at home were significantly lower than those in the reference population, which suggests that the parents experience stress even when the child is healthy and has been discharged.

At home, the parents in this study had returned to their daily lives and may have been struggling to cope with parental stress in combination with work stress. This could explain the increase in their morning awakening cortisol compared with their awakening cortisol levels at the paediatric ward. Hibel, Mercado, and Trumbell (2012) have shown that parental stress alone is not sufficient to physiologically arouse mothers. High self‐rated parental stress, in combination with stress from another life domain, for example work, increases the morning awakening cortisol levels in mothers of healthy children on workdays above the level of non‐workdays (Hibel et al., 2012). This needs to be further investigated, since high levels of cortisol during a prolonged period may have negative consequences for, for example, cognitive functions (McEwen & Sapolsky, 1995).

Cortisol awakening response was not lower in the hospital, which is incompatible with our hypothesis. This result shows that the parents were able to respond with a healthy increase in the postawakening cortisol response. In other words, the parents were not exhausted or worn out while accompanying their sick children at the family‐centred paediatric wards.

The strength of the study presented here is that paired statistics were used to compare parents’ cortisol levels at the hospital with those after discharge. The sampling procedure was consistent with the expert consensus guidelines for assessment of saliva cortisol response (Stalder et al., 2016). Moreover, the study gives a first overview of the HPA axis activity in parents accommodated with their sick child in family‐centred paediatric wards and can be used as a foundation for future research.

## 7 LIMITATIONS

Our results should be interpreted in the light of several limitations. Five parents at the paediatric ward and eight parents at home had a negative CAR, with higher morning awakening cortisol than postawakening cortisol. It is possible that the timing of sampling was inaccurate. The parents were instructed to take the saliva sample immediately on awakening. However, 5‐min delay is enough to negatively affect the analysis of CAR (Smyth, Clow, Thorn, Hucklebridge, & Evans, 2013; Smyth, Thorn, Hucklebridge, Clow, & Evans, 2016) and such a delay may have occurred if the parents were occupied caring for their child or if they set the alarm clock to allow a snooze. Moreover, the study design could have been strengthened by asking the participants to use a self‐rating stress scale in conjunction with the cortisol sampling.

## 8 CONCLUSION

Parents of sick children are exposed to stressors, which leads to lower morning awakening cortisol levels in the hospital compared with at home after discharge. The levels are also lower than those of a reference population. After discharge, the morning awakening cortisol levels remained lower than those of the reference population, which indicates that the parents were exposed to stressors also at home. Since this is the first study, more research is necessary to obtain deeper knowledge of how and why the hospital stay affects the parents’ cortisol levels. Parents’ stress when the child is sick need to be more acknowledged because their constant support is crucial for the child and moreover to decrease the risk of stress‐related consequences in parents of sick children.

## CONFLICTS OF INTEREST

The authors have no conflicts of interest to declare.
